# Severe intermittent anxiety attacks: A diagnosis challenge in temporal lobe epilepsy

**DOI:** 10.17712/nsj.2017.1.20160284

**Published:** 2017-01

**Authors:** Abdulaziz T. Alshomrani, Radwan M. Zaidan, Yosef T. Abdulmalik, Nader M. Alrahili

**Affiliations:** *From the Department of Clinical Neuroscience (Alshomrani, Zaidan, Abdulmalik, Alrahili), College of Medicine, Al Imam Mohammad Ibn Saud Islamic University, and from Sabic Psychological Health Research and Applications Chair (Alshomrani), College of Medicine, King Saud University, Riyadh, Kingdom of Saudi Arabia*

## Abstract

Psychiatric symptoms are frequently reported with epilepsy. Anxiety symptoms are the most common psychiatric expressions of temporal lobe epilepsy (TLE). Longer duration of the epileptic manifestation can be mistaken for psychiatric diseases, particularly when psychiatric symptoms are the only manifestations of the disorder. Here we introduce a case of a 27-year-old Saudi man presented to our clinic with a history of sudden and severe anxiety attacks over the prior 2 years, each lasting for 2-3 days. The attacks recurred monthly without clear triggers, and he recovered his normal clinical state between them. His condition worsened with antidepressants and improved with antiepileptic. Later follow ups and work ups supported the diagnosis of temporal lobe epilepsy. Diagnosis after such presentation may be challenging and we tried, in this case, to enhance awareness of such an unusual presentation.

Psychiatric disorders, including anxiety symptoms and suicidal thoughts, are more frequently reported with epilepsy than general population and could be underdiagnosed.^1^ Refractory focal temporal lobe epilepsy (TLE) has been reported to be more frequently associated with anxiety disorders particularly at a younger age and with shorter duration attacks.^2^ Anxiety symptoms such as fear, anger, and irritability represent the most common psychiatric expressions of simple focal temporal lobe attacks.^3^ The duration of the ictal phase is usually brief (1 to 3 minutes or less). However, a longer duration of the epileptic manifestation is defined as non-convulsive status epilepticus, and the diagnosis can be mistaken for psychiatric diseases, particularly when psychiatric symptoms are the only manifestations of the disorder.^4^

Psychiatric disorders may not be directly connected to the seizure activity (inter-ictal), and they may appear before or after the diagnosis of epilepsy.^5^ Notably, controversy continues to surround the phenomenology of psychiatric disorders in epilepsy and whether the symptom profile and semiology are comparable to psychiatric disorders in the general population (namely, DSM-IV or ICD-10).^5^

Hippocampal sclerosis is the most common feature of TLE, causing the most frequent epileptic disorder. The morphological changes in the horne d’Hammon are the most characteristic of the condition.^6^ Regional atrophy with loss of neurons may vary in severity from an area to another. However, it is not the only feature, and more or less severe gliosis may be associated with the neuronal loss leading to abnormal neuronal activity and epilepsy network that involve structures belonging to a visceromotor system functionally associated with mood, emotions, and visceral reactions to emotional stimuli.^6^

The importance of the amygdala as a part of a neuronal network which dysfunction play an important role in the generation of anxiety and epileptogenesis has been highlighted in previous research works.^7^ In this regard, Lanteaume et al^7^ have shown the generation of negative emotions such as fear, anxiety and sadness by direct electrical stimulation of the amygdala, particularly on the right side. On the basis of these data, it may be hypothesized that recurrent stimulation of the amygdala during temporal lobe seizures may cause increased irritability of this region interictaly, which becomes clinically manifested as an anxiety disorder.^7^

We report the case of a young Saudi man with a 2-year history of recurrent attacks of severe anxiety lasting 2 to 3 days with complete recovery between attacks and improvement after antiepileptic treatment. The importance of this case is derived from its diagnostic difficulty and the need for increased awareness of its presentation.

## Case Report

**Figure 1:**
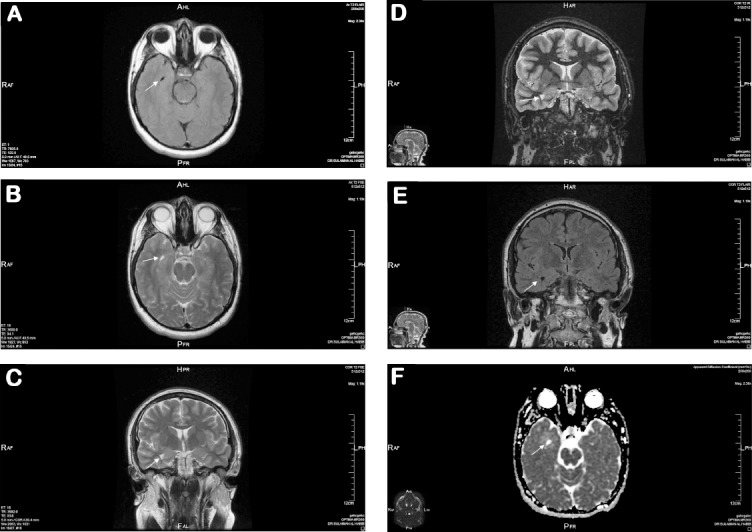
- Showing right temporal trophic changes with dilated right temporal horn **a)** FLAIR axial MRI, **b)** T2 FSE weighted axial MRI, **c)** T2 FSE weighted coronal MRI, **d)** T2 IR weighted coronal MRI, **e)** FLAIR coronal MRI, **f)** Diffusion weighted coronal MRI.

A 27-year-old Saudi man presented to our clinic in 2011 with a 2-day history of severe anxiety, fearfulness, and tremors that kept him home-bound and dependent on his family for the majority of his needs. He was unable to attend college or pray in Masjid. He reported a history of sudden, severe anxiety attacks over the prior 2 years, each lasting for 2 to 3 days. The attacks recurred monthly without clear triggers. He reported a bad and vague burning-clothes-like smell during the attacks, and he recovered his normal clinical state between them. One year earlier, he consulted a psychiatrist, who prescribed 40 mg paroxetine once daily. However, his condition deteriorated, and he began experiencing attacks twice monthly. He denied a family history of mental illness, but a cousin was diagnosed with epilepsy. The patient denied drug misuse. Examination revealed anxiety, fearfulness and postural tremor with no other positive neurological findings. His thinking and cognition were normal. His brain MRI showed trophic changes on the left temporal lobe with a more patent temporal horn (**Figure 1**). The inter-ictal EEG at this time (patient was symptom-free) did not show abnormalities.

**Figure 2:**
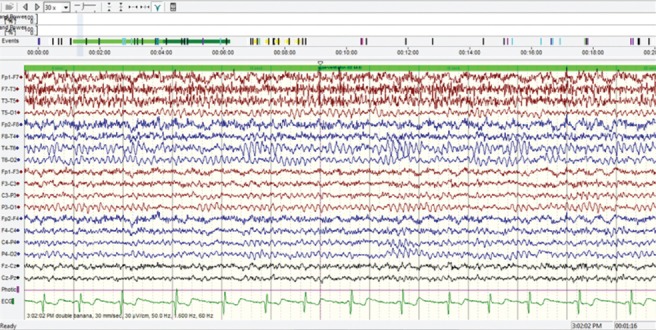
- Interictal EEG: longitudinal montage (double Banana) showing no clear epileptiform activity.

**Figure 3:**
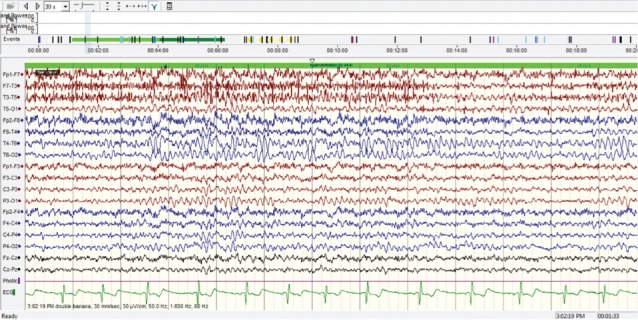
- Interictal EEG: longitudinal montage (double Banana) showing no clear epileptiform activity.

Clinical findings suggest epileptic attacks with dys-osmic expression. Paroxetine was discontinued, and the patient dramatically improved (no more attacks) on 400 mg carbamazepine twice daily. This was continued for more than a year. The patient returned for follow up after a 14-month absence. He reported recurrent brief anxiety attacks after tapering the carbamazepine to 600 mg daily and starting paroxetine at 20 mg daily at the instruction of another physician. One attack was similar in severity to those described at the initial presentation. A repeated EEG failed to show epileptiform activities (**Figures 2-3**). He refused long-duration EEG recording using an epilepsy monitoring unit. His condition completely remitted for one year on 1200 mg carbamazepine daily and discontinuation of paroxetine.

## Discussion

Attacks of anxiety associated with seizure and attacks of epilepsy with pure psychiatric clinical manifestations continue to challenge physicians and can easily be missed.^5^ Clinical findings alone may not be sufficient to confirm the diagnosis of prolonged attacks or, eventually, non-convulsive status epilepticus.^5^ Anxiety, a common presentation in a psychiatric setting, was the only psychiatric manifestation in our case in the form of attacks lasting 2 to 3 days. Such presentation may not be easy to differentiate from a simple psychiatric condition. The olfactory symptom was the main clinical finding that might suggest epileptic nature.^3^ The lack of a specific EEG finding might be the reason behind the diagnostic delay. Although MRI findings might attract the attention on the nature of the clinical setting. During the course of the disorder, the deterioration of the patient’s condition on Paroxetine, a specific serotonin reuptake inhibitor (SSRI) that may lower the epilepsy threshold as claimed in some studies^8^, could have been another reason to consider a different diagnosis. Correlating multiple findings during the course of the setting, particularly the excellent clinical response to carbamazepine, the olfactory hallucination, the positive family history of epilepsy rather than mental disorders, the MRI finding, and the deterioration on Paroxetine supported the diagnosis of TLE.

Ictal anxiety with fear feelings usually occur over a period of seconds to minutes in simple focal temporal seizure with or without the loss of awareness in the formerly termed complex focal seizure.^4^ Ictal anxiety disorders may not be easily differentiated from panic disorders, yet it could be the only clinical presentation.^3^ On the other hand, psychiatric symptoms, including anxiety and/or depression, may be epilepsy related following the attacks of generalized or focal epilepsy (post-ictal).^9^ Such a possibility is likely in our case and this is supported by the lack of epileptiform discharges on the second EEG carried out during the episode. However, it was performed at the end of an attack. An electrical attack would indicate the pure epileptic nature of the anxiety symptoms.

Anxiety may be an interictal manifestation in approximately two-thirds of patients with epilepsy. It may last for hours or days during the postictal period and it is usually associated with dys-autonomic symptoms such as tachycardia, sweating, and dyspnea.^9^

Unfortunately, our patient lost the opportunity to have optimum EEG recordings during his attacks. The changes on the MRI could explain the underlying cause of our patient’s ictal and postictal events in view of recent data suggesting that ictal fear with coordinated behavior and autonomic features may be part of a complex information-processing neuronal network involving temporal cortex and the well connected structures to it including limbic, orbito-prefrontal and anterior cingulate cortices.^10^

In conclusion, although psychiatric clinical ictal expressions have been, and continue to be, reported, diagnosis after such presentation may be challenging. Yet any delay may dramatically affect the patient’s quality of life. We illustrate in this report the difficulties in diagnosing TLE-related anxiety disorders and thereby contribute to enhancing awareness of such an unusual presentation.
